# Complete genome analysis of human influenza C virus co-infection with WU polyomavirus in a Sri Lankan child: A brief report

**DOI:** 10.1099/acmi.0.000968.v3

**Published:** 2025-10-29

**Authors:** T. T. Pattiyakumbura, I. De Silva, A. Bowatte, S. Perera, D. Rathnayake, H. D. W. S. Kudagammana, M. A. R. V. Muthugala, T. K. G. S. Sumathipala

**Affiliations:** 1 Department of Clinical Virology & Genomics Laboratory, National Cancer Institute, Sri Lanka 10280, Sri Lanka; 2 Postgraduate Institute of Medicine, University of Colombo, Colombo 00700, Sri Lanka; 3 Teaching Hospital, Peradeniya 20400, Sri Lanka; 4 Department of Microbiology, Faculty of Medicine, University of Peradeniya, Peradeniya 20400, Sri Lanka; 5 Medical Research Institute, Colombo 00800, Sri Lanka

**Keywords:** influenza C, respiratory tract infection, Sri Lanka, whole-genome analysis, WU polyomavirus (WUPyV)

## Abstract

**Background.** Influenza C virus (ICV) is a lesser known member of the *Orthomyxoviridae* family, primarily causing respiratory tract infections in children. Co-infection with WU polyomavirus (WUPyV), a recently identified human polyomavirus, has been rarely reported. This study presents the first laboratory-confirmed case of ICV infection in Sri Lanka and its co-infection with WUPyV.

**Methods.** Nasopharyngeal and oropharyngeal swabs were collected from children aged 3 months to 14 years with respiratory tract symptoms between November 2022 and February 2023. Samples were screened using multiplex real-time PCR (RT-PCR) and Severe Acute Respiratory Syndrome Coronavirus 2 (SARS-CoV-2) RT-PCR. A nasopharyngeal swab from a 14-month-old infant showing an insignificant curve in respiratory PCR was subjected to whole-genome sequencing using the Illumina platform. Data were analysed for genomic characterization, and phylogenetic analysis was performed using the haemagglutinin-esterase gene of ICV.

**Results.** Full-genome sequencing identified ICV and WUPyV in the sample. Phylogenetic analysis revealed that the ICV isolate belonged to the C/Sao Paulo lineage. The patient presented with mild symptoms, including fever, cough and cold, with normal inflammatory markers, and recovered with supportive care.

**Discussion.** This case highlights the importance of considering ICV in paediatric respiratory illnesses, despite its under-diagnosis due to limited diagnostic tools. Unlike influenza A and B, ICV lacks neuraminidase, rendering neuraminidase inhibitors ineffective. The absence of ICV in current influenza vaccines further complicates preventive strategies. Co-detection of WUPyV raises questions about its role as a co-pathogen, with its clinical significance requiring further investigation.

**Conclusion.** This report underscores the need for enhanced molecular diagnostic techniques and surveillance to better understand the epidemiology and clinical impact of ICV and its co-infections.

## Data Summary

The data presented in this study form the foundation for the conclusions drawn and enable the replication of the methodologies described. The GenBank accession number for the complete WU polyomavirus genome is PQ066767.1 (https://www.ncbi.nlm.nih.gov/nuccore/PQ066767.1).

## Introduction

Influenza viruses are significant human pathogens due to their ability to cause annual epidemics that result in morbidity and mortality in thousands of people worldwide, also occasional, but severe, global pandemics. Influenza viruses are the sole members of the Orthomyxoviridae family. Four immunological types of influenza viruses are known: A, B, C and D. Only influenza A, B and C are known to infect humans. Influenza C virus (ICV) was first identified in 1950; however, it is less well-described than influenza A and B viruses in humans [1]. There are several differences between influenza A, B and C viruses. ICV has nine viral proteins, whereas influenza A and B have 10 and 11 viral proteins, respectively. Unlike influenza A and B, ICV lacks the neuraminidase outer-membrane protein. These differences contribute to its unique disease characteristics and are significant for antiviral treatment considerations. Like influenza B, ICV does not undergo reassortment and antigenic shift, which differs from influenza A. However, ICV does experience antigenic drift, allowing multiple variants to circulate simultaneously. Like other influenza viruses, ICV circulates primarily from winter to early summer in temperate countries. In addition to human infections, ICV has been identified as a cause of illness in swine and dogs. ICV has been rarely identified as a cause of medically attended infection in adults, but it has been identified as a cause of respiratory tract infection in children [2]. In a hospital-based study in Spain, ICV was detected in ~0.9% of infants admitted with respiratory infections, with some cases occurring as co-infections alongside Respiratory Syncytial Virus (RSV) or adenovirus, indicating its role among the viral causes of hospitalized illness in young children [3]. More recently, sentinel surveillance in Austria during 2022 demonstrated an increased detection of ICV in both children and adults compared with previous years, suggesting that ICV activity may fluctuate over time and become more prominent during certain influenza-like illness seasons [4].

The lack of readily available laboratory diagnostics may have underestimated the true disease burden of ICV infection. This communication presents a detailed genomic analysis and characterization of the first laboratory-confirmed human ICV infection in Sri Lanka.

## Methodology

This study was initially conducted as a hospital-based cross-sectional investigation, collecting nasopharyngeal and oropharyngeal swabs from children aged 3 months to 14 years who attended paediatric wards with signs and symptoms of respiratory tract infections in two main hospitals in the central part of the country between November 2022 and February 2023. These samples were tested for potential infectious agents using a respiratory multiplex real-time PCR (RT-PCR; RespiFinder^®^ 2Smart; PathoFinder B.V., Netherlands), which can simultaneously detect 20 viral and 4 bacterial agents through melt-curve analysis, and also for the detection of SARS-CoV-2 RNA by locally validated commercial RT-PCR kit (Bioneer; Bioneer Cooperation, South Korea). Of the 75 specimens tested, 62 were positive for one or more identifiable infectious agents. Among the positive specimens, one nasopharyngeal swab showed an insignificant amplification curve with no identifiable pathogen. This sample was subsequently subjected to genome sequencing (Illumina NextSeq 1000 platform; Illumina, Inc., USA). The genome was successfully amplified and sequenced, followed by data analysis and the construction of phylogenetic trees to investigate its genetic characteristics.

### Specimen analysis

#### RNA extraction and full-genome analysis

RNA was extracted from 200 µl of viral transport medium containing nasopharyngeal and oropharyngeal swab samples using the Maxwell^®^ automated total nucleic acid extraction system (Promega Corporation, USA), according to the manufacturer’s protocol. Library preparation was performed, and genome sequencing was carried out using the Illumina NextSeq 1000 platform (Illumina, Inc., USA) with the Illumina Respiratory Pathogen ID/AMR Enrichment Panel (RPIP) kit. The resulting sequence data were analysed using the Explify RPIP Data Analysis Pipeline and a metagenomic analysis pipeline in the Illumina BaseSpace Sequence Hub to identify viral pathogens [5]. Complete genomes for both ICV and WU polyomavirus (WUPyV) were derived from the sequence data of the sample.

#### Quality control and quality assurance measures

Quality assurance measures were implemented throughout the procedure to ensure data integrity and reliability. Pre-extraction, specimen integrity was maintained during collection and transportation to prevent degradation. During RNA extraction, extraction-negative and -positive controls were run in parallel to monitor contamination and verify extraction efficiency. For library preparation, the concentration of prepared libraries was measured using fluorometric quantification (Qubit) to ensure optimal input for sequencing. DNA fragment length analysis was performed to ensure that the fragments were within the specified size range as outlined in the library preparation protocol. During data sequence analysis, quality filtering was applied to remove low-quality reads and trim adapter sequences, ensuring that the remaining reads were of high quality for accurate downstream analysis.

## Results

### Clinical presentation of the patient

This nasopharyngeal swab was taken from a 14-month-old infant who had been experiencing a cough and cold for 4 days, along with a fever that began 1 day prior. His lungs were clear, and oxygen saturation was maintained at 99% on room air. Due to a documented fever of 101 °F, by hospital policy, the child was admitted to the paediatric ward for 24 h observation and care. However, his full blood count and inflammatory markers were normal (C-reactive protein level is less than 6 mg dl^−1^). He was discharged after showing no signs or symptoms of respiratory distress and was provided with supportive medications. The nasopharyngeal swab collected on the fourth day of illness, which initially showed an insignificant curve in the respiratory multiplex PCR assay, was further subjected to full-genome sequencing. It was subsequently identified as ICV RNA and WUPyV.

### Phylogenetic analysis of the ICV

**Fig. 1.**
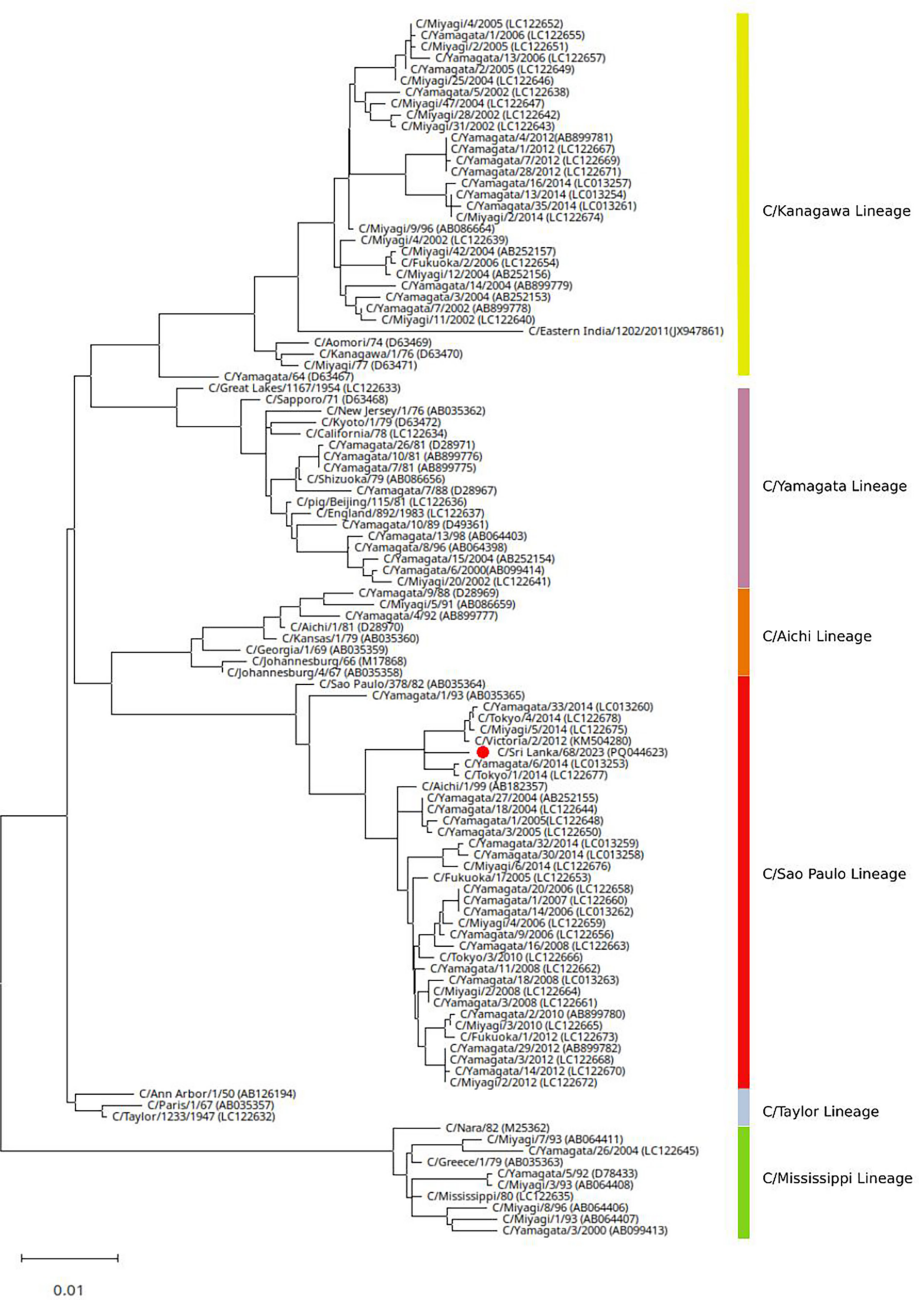
Phylogenetic tree of ICV based on the HE gene.

Phylogenetic tree construction was performed using the haemagglutinin-esterase gene of ICV, inferred using the maximum-likelihood method and Tamura–Nei model. The tree construction used a known list of ICV sequences representing all six lineages. C/Sri Lanka/68/2023 isolate is clustered within the C/Sao Paulo lineage. The GenBank accession numbers for all seven segments of the ICV genome are as follows: segment 7 (PQ044626.1), segment 6 (PQ044625.1), segment 5 (PQ044624.1), segment 4 (PQ044623.1), segment 3 (PQ044622.1), segment 2 (PQ044621.1) and segment 1 (PQ044620.1) [6,12]. The GenBank accession number for the complete WUPyV genome is PQ066767.1 [13]. Additional analyses were performed using advanced bioinformatics tools (Fig. 1).

## Discussion

Full-genome analysis and sequencing revealed that the patient was infected with ICV. To the best of our knowledge, this was the first laboratory-confirmed case of human ICV infection in Sri Lanka. Phylogenetic analysis of the specimen further showed that this isolate is clustered within the C/Sao Paulo lineage.

Like other influenza virus types, ICV is a negative-sense RNA virus, belonging to the Orthomyxoviridae family, and it has been identified as a causative agent of respiratory tract infection in children. Although it is less well-studied compared with other influenza viruses, ICV infection appears to be common. Community-based serological studies indicate that the prevalence of influenza C-specific antibody levels varies between 60% and 100% [14,15]. However, the presence of influenza C-specific antibodies does not confer complete protection, as serological evidence of past exposure can still develop symptoms and shed viruses [16]. One potential explanation for this phenomenon could be the circulation of at least six distinct lineages of ICVs [17]. However, the mechanisms by which these lineages evolve and co-circulate within a population remain poorly understood. Given that, ICV has been shown to cause a spectrum of symptoms, especially in children, diagnosis of ICV should be considered in the range of viral aetiologies that cause respiratory illness [18]. However, as the samples tested were restricted to patients who visited the hospital, the prevalence of ICV respiratory illness in the community remains unclear. Also, when considering the treatment for ICV infection, unlike influenza A and B, ICV lacks the neuraminidase outer-membrane protein. This distinction holds significance for antiviral treatment considerations, as neuraminidase inhibitors may prove ineffective in treating ICV infections. Additionally, the currently available influenza vaccine does not include the influenza C strain. Consequently, vaccination may also be ineffective as an infection-prevention strategy against ICV.

Although different approaches are currently available for the diagnosis of influenza A and B infections in humans, including viral isolation in cell culture, immunofluorescence assays, nucleic acid amplification tests and immunochromatography-based rapid diagnostic tests, no such tests exist for ICV. The under-diagnosis of ICV has resulted from difficulties in culturing this virus and the lack of readily available monoclonal antibodies for detection by direct fluorescence microscopy. Although cell culture methods have been used for the isolation of ICV, the rate of recovery is low [19]. Amniotic inoculation of embryonated hen’s eggs remains the most sensitive technique to isolate this virus; however, few clinical laboratories have this facility. The implementation of molecular diagnostic techniques in research and surveillance settings has the potential to substantially advance the understanding of the spectrum of ICV infection [20]. This, in turn, could facilitate the development of targeted treatment strategies and preventive measures.

In addition, full-genome sequencing identified WUPyV as a co-pathogen in the patient’s sample. Currently, 13 species of polyomaviruses are associated with human infections, with BK Polyomavirus (BKV), JC Polyomavirus (JCV), KI Polyomavirus (KIV), WUV and Merkel Cell polyomavirus being the most prevalent. WUPyV was recently discovered in the respiratory secretions of children with acute respiratory symptoms [21]. Seroepidemiological studies indicate that WUPyV, like other polyomaviruses, infects individuals early in life, likely through respiratory and faecal–oral routes. These viruses remain latent in various body tissues and can reactivate under immunosuppressive conditions [22]. A definitive link between WUPyV and respiratory disease has not been established due to multiple factors, including the lack of specific clinical or radiological indicators of the infection, frequent co-detection with other respiratory pathogens, virus detection in asymptomatic individuals and variability in viral load measurements. Also, polyomaviruses are notably stable because they can withstand harsh environments, including sewage and temperatures exceeding 95 °C. Their robust nature and stable double-stranded circular DNA genome allow them to persist in detectable states within environmental and clinical samples for extended periods [23]. Therefore, clinical interpretation should be approached with caution.

The co-detection of ICV and WUPyV in this patient raises the question of whether viral interactions may influence host susceptibility or disease severity. Although polyomaviruses are known to persist latently and can reactivate under conditions of immune modulation, their exact role in influencing host immune responses during co-infection with respiratory viruses such as ICV remains unclear. Some studies suggest that co-infections may alter local immunity, facilitating viral persistence or increasing the likelihood of symptomatic illness; however, definitive mechanisms have yet to be established[23].

## Abbreviations

BKV: BK polyomavirus
HE: haemagglutinin-esterase
ICV: influenza C virus
JCV: JC polyomavirus
KIV: KI polyomavirus
RPIP: Respiratory Pathogen ID/AMR Enrichment Panel
RSV: Respiratory Syncytial Virus
RT-PCR: real-time PCR
SARS-CoV-2: Severe Acute Respiratory Syndrome Coronavirus 2
WUPyV: WU polyomavirus
